# Supplementary material for: Related adjustment of firm production after demand shocks
Source: PLoS One. 2023 Jan 18;18(1):e0280461. doi: 10.1371/journal.pone.0280461 (PMC9847954; doi:10.1371/journal.pone.0280461)
Supplement: Attachment — Submitted filename: Response to the Reviewers Plos1_0630.docx [file pone.0280461.s001.docx]

**Response to the Reviewers**

Dear Editor and Reviewers,

We appreciate the opportunity to revise and resubmit our paper “Related and unrelated firm diversification in crisis and in prosperity”. We are thankful for the comments of the reviewers that enabled us to develop our paper more solid theoretically and empirically. We have revised the manuscript along two major lines. First, we reframed our theoretical approach that better corresponds to our empirical strategy, and second, we included an important factor to the analysis suggested by both reviewers, the liquidity constrains. We aimed to respond to each reviewer comments and implement them to the best of our knowledge. We are addressing each of the concerns and comments raised step by step in the followings.

**Reviewer #1**

Major concerns:

A) The nature of the crisis and its effects on the product portfolios. The authors explicitly assume that the crisis in 2008-2009 affected firms through a demand shock, hence they empirically measure the intensity of the shock over time by looking at drops in sales (market shrinkage). Actually, the global crisis in 2008-2009 had its roots in shocks that propagated from financial markets, thus affecting the real economy through credit shortages for both firms and consumers. Thus, demand dropped as a response to a financial shock. In this context, the correlation between market shrinkage and product portfolios in the paper could be a spurious one. I suggest the author to control explicitly for heterogeneous financial exposure of firms and their impact on the changing composition of product portfolios. Market shrinkages can be the result rather than the driver of changing product portfolios during a crisis in 2008-2009. After testing explicitly for the specific nature of the crisis in 2008-2009, the authors could streamline better their hypotheses in the framework they propose.

*Thank you for drawing attention to this important issue. We included this argument to Section 2.3. regarding theories. This also made us modifying H1, breaking it down into H1a about the impact of negative demand shock in general, and H1b about the impact of negative demand shocks coupled with high financial exposure. Correspondingly,* *we included the financial exposure variable (together with its interaction with negative demand shocks) throughout the models in the paper.*

B) Empirical strategy. The choice of the empirical strategy is most problematic. What the authors actually test is not the change of product portfolios by separating times of crisis from times of prosperity. (The ambiguity starts in the title, when they mention both “times of crisis and prosperity”.) As they implement a panel fixed effects with both firm and time dummies, the coefficients they find can only be interpreted as average effects along the entire period they analyze, 2005-2012, thus confounding both years of crisis and so-called prosperity. (Please note that markets can be volatile also in times of overall ‘prosperity’.) In this case, I suggest implementing an event study to check if there is actually an apparent impact on the combination of product portfolios in times of crisis that is different from regular times. In this case, see also the specific comment on the nature of the crisis from point (A), and its economic mechanism having an impact on firms’ decisions.

*Thank you for raising our attention to this issue. After reading your comments and re-reading the paper, the discrepancy between the introduction and the empirics also became apparent to us, that is based on the title, and the arguments in the introduction (and partly also in the theory), the reader would expected an empirical strategy that compares firm behavior over different time periods.*

*To resolve this issue, we decided to fit the introduction and theory part to the empirics, and not the other way around. We did so, as we believe that a key contribution of our paper is the idea that market trends can be quite different across product markets, and those can be utilized for identification of firm behavior. In case we compared different time periods, we could not utilize this heterogeneity, and would loose both in terms of empirical validity and statistical power.*

*According to this, we re-framed the introduction, removing arguments about “times of crisis”, but emphasizing negative demand shocks that firms experience. In the theory section most arguments remained valid in this framing, but the ones about the strategies of firms in economic crisis we reconsidered. We also modified the title of the paper.*

C) Technological relatedness. The authors mainly rely on a network measure proposed in an unpublished working paper by Neffke and Henning in a working paper series. The main problem of that measure is that it does not actually catch direct technology relationships. It assumes that if products are produced by the same firms, then they are likely related in productive technologies needed to produce them. Such a measure is a sort of ‘revealed technology relatedness’, which is possibly biased by the presence of: 1) conglomerated firms, which decide to differentiate their production lines in structurally unrelated markets; 2) vertically-integrated firms, which decide to produce the inputs they need for their output. The suggestion is to discuss these limits and possibly implement an augmentation of the binomial regression model of eq. 1, to take care of the above-mentioned biases. Right now, it is not clear to me what is the economic role of the covariates of eq. 1 (number of active firms, average profit level, income level, firm size in employees, and value added).

*This method does indeed measure a revealed technology relatedness, which is however a fairly common and accepted method in the literature if we cannot directly measure technology relationships. The description of the Neffke and Henning method has been published in several later applications in peer-reviewed journals (see e.g. Neffke F, Henning M., Boschma R. (2011): The impact of ageing and technological relatedness on agglomeration externalities: a survival analysis. Journal of Economic Geography, 12(2): 485-517.; Neffke F., Henning M., Boschma R. (2011): How do regions diversify over time? Industry relatedness and the development of new growth paths in regions. Economic Geography 87(3):237-265.; Neffke F., Henning M., Boschma R., Lundquist K.J. & Olander L.O. (2011). The dynamics of agglomeration externalities along the life cycle of industries. Regional Studies 45(1): 49-65.), but the most detailed description can be found in the referenced working paper, so it is usually referred to.*

*Presence of conglomerated and vertically-integrated firms would not be a problem if we had site-level data, as firms produce technologically related products at the same site, which can benefit from the resulting cost efficiency in production. Unfortunately, our database only contains firm-level data. However, two things reduce the problem raised. First, it is precisely to avoid this multi-plant problem that we are looking at manufacturing industries. Békés and Harasztosi (Békés, G. and Harasztosi, P, 2013. "Agglomeration premium and trading activity of firms," Regional Science and Urban Economics, Elsevier, vol. 43(1), 51-64.) show that in the Hungarian manufacturing industries over 90% of firms have one site only, which suggests that the bias is not significant. Moreover, as shown in the description of the method, the difference between the predicted and observed co-occurrences reveals the relatedness between each industry pair. The relatedness indicator between two industries would be significantly distorted if both industries were composed of very similar conglomerates or vertically integrated companies expanding in the same unrelated directions. Thus, the problem is considered legitimate, but the arguments above suggest that the bias does not really give cause for concern. Nevertheless, we thank for drawing our attention to this necessary addition, and we have included a discussion of this bias in the description of the method.*

*Regarding the economic role of the covariates in equation 1, as we discussed above the equation, diversification into products can be motivated not only by economies of scope and technological proximity. Other factors, such as high expected profits or industry size are important as well so that firms might decide to expand their activities to attractive industries. To control for other factors that influence the number of co-occurrence links we followed Neffke and Henning (2008) and estimate the expected number of co-occurrences for each pair of industries using industry specific characteristics as predictors (e.g. profitability, sales volume, competition expectations, wage level etc.).*

D) External validity. The framework the authors adopt implicitly considers any economic crisis as having the same impact on product portfolios. I think it would be useful to comment if such a framework could be valid when one confronts with economic crises that have a different origin. Can findings related to a financial crises be extended to other sorts of crises, like the latest pandemic crisis? Can the search for different (foreign) markets prevail, hence bringing more diversification in products, when the crisis is more national and less global in nature?

*We examine the impact of demand shocks in general, which can arise for several reasons. In the period under study, there were several such causes, e.g. EU accession, import shocks and financial crisis, all of which have in common that they cause a demand shock. However, in case of financial crisis there is an additional mechanism, which we have examined separately in the new version. We tried to clarify this in the paper. And yes, all of this makes us think that our results can apply to any crisis that causes a demand shock.*

Minor comments:

- The paper in general reads well, but it lacks conciseness starting from the abstract. It is not immediately clear what the research question is. There are many statements or claims that are not actually investigated in the paper (e.g. “what conditions drive related versus unrelated diversification of firms, is still poorly understood.”). The conclusions even relate the work to pieces of literature that were not discussed in the literature review, e.g., trading firms and trade policy, although the paper does not specifically investigate exporters or importers. I suggest cutting unnecessary digressions.

*Thank you for your suggestion. We have tried to clarify the research questions and focus our message better.*

- Some descriptive statistics of the sample could help. How many firms in each industry? How representative they are of the entire population? I understand that the same data have been often used in other published economic papers. Please provide references for data validation.

*We inserted data to the text in section 3.1, and also a new figure (Figure 2) on the distribution of firms by size and industry, and comparison to official publication (Eurostat).*

- Please provide a better resolution of graphs.

**Reviewer #2**

i) The authors selected the period 2005-2012 whereas the PRODCOM database covers the peri**od 2003-2012** so the authors may provide a brief explanation about the sample construction choices.

*Thank you for pointing this out. In fact we utilize the data for the analysis from the whole period of 2003-2012; so we modified the description accordingly. What might have lead to this mistake in the description is that we use both a lagged and forward term in the estimation, which decreases the number of effective years used for identifications by two.*

ii) It is interesting to better point out the number and type of booms and busts that you observe in the period of analysis as these market fluctuations may be attributed to different stimuli that are likely to affect industries/firms in a different way.

*Thank you, we added a related description to the Introduction (at line 100).*

Empirically, the paper will also benefit from providing robustness to alternative operationalizations of economic crisis through measures that best capture shift in demand for example.

At page 24, the authors argue that Coherence of the previous year is still the strongest predictor of Coherence at time t, I was then wondering whether and how the economic crisis could really play a role, as so far does not seem to be a strong predictor and this may require a realignment between the theory and the empirics.

*Thank you for this comment. We modified the description of the results here, removing the text that previous year was the strongest predictor – which is in fact true statistically, however this does not have theoretical relevance, as the coherence of previous year is only a technical variable to serve as reference point for the next year and to capture the stability of the process.*

The fact that in Table 4 and 5 Market Shrinkage and Import Shock have opposite sign may also require **additional explanation/interpretation**.

*At the suggestion of the other reviewer, we included a financial exposure variable (together with its interaction with negative demand shocks) throughout the models. This changed the results slightly, but there was still a difference between the effects of the Market Shrinkage and Import Shock variables. This difference is explained in more detail on page 33 and also in Conclusions on pages 37-38.*

At page 17, the authors highlight a potential problem of endogeneity which probably call for additional explanations.

*We have supplemented the explanation.*

About the Import measure, I was wonder whether you observe increasing import competition in general and not only during a crisis. If that is the case what you observe with this variable may not be related to a crisis event, this would probably require some additional explanations.

*Import pressures are another possible source of demand shocks in product markets, measured over the entire time window under study. We have tried to clarify this at several points in the paper.*

iii) It is also interesting to know more about how many firms are included in the sample and what are their characteristics for example in terms of size, R&D expenditures, age, diversification over the period, performance indicators.

*In the new version we included the information on distribution of the firms included in terms of size and industry to section 3.1 (and Figure 2). Unfortunately, our data do not cover R&D expenditures and the age of firms, so we could not include these. We also supplemented the data description with information on size and performance indicators (Table 1 in section 3.1)*

Size appears to be a very important characteristics as if your sample include mostly small companies, it is rather trivial that their best choice is go for efficiency solutions and opt for a coherent product portfolio since they are not deep pocket and suffer from liability of smallness. In this line, it is interesting to provide some measure about slack resources or financial constraints of these firms which may also be used as controls in the regressions.

*Thank you for this suggestion. In line with this suggestion, and with the related one of Reviewer 1 to address financial constraints directly, we added the analysis of financial constraints to the analysis throughout the article, and also modified H1, breaking it down into two sub-hypotheses; H1a about the impact of negative demand shock in general, and H1b about the impact of negative demand shocks coupled with high financial exposure.*

iv) The authors also point out, at page. 7, that extant literature showed that in R&D intensive industries unrelated diversification is frequently applied therefore I wonder whether the authors experiment with the regression by using R&D intensity as a possible control.

*Thank you for this suggestion. However, we could not implement this, as we do not have data about R&D expenditures.* *(R&D data would only be available from the European Innovation Survey, but that considers a more limited sample that only partly overlaps with the Prodcom.)*

v) At page 14, the authors make an example about the products in calculating the technological relatedness, what if the same product is in two industries? Are these cases possible or frequent?

*In our approach it is not possible for a product to belong to two different industries, as we assign each firm the industry, to which its core product belongs to (when the industry is defined by the 4 digits of the 6-digit product code). As the product classification is hierarchical, it is not possible that the same product code appears under two separate industry codes. We describe this procedure in paragraphs 2-4 of section 2.3. Note that with this method we in fact disregard the registered industry code of the firms, to which they classified themselves; and “correct” that based on their actual production behaviour.*

I would also include a real case example picked from your sample.

In general, when describing data and measure used it would be good to provide some examples. For instance, by picking up some companies including in your sample (eventually in an anonymized way) and provide some descriptive for the focal firm or informing the readers about the exact variables available at PRODCOM.

*Thank you, we agree that this would indeed be informative. However, we are unable to do that due to the explicit restriction in our contract with the Central Statistical Office that forecloses reporting any information that refers to the activity of a single individual unit (firm), even in an anonymised way.*

Theory: from a theoretical point of view I would include the discussion on opportunity cost looking for example at the work by (Aghion and Saint-Pauk, 1998; Berchicci et al., 2013; Steenkamp and Fang, 2011). (opportunity cost vs. financial cost strand of literature) in contrast with the financial constraint argument.

*The proposed line of literature gives the same cost-effectiveness argument, starting from the same literary roots (Penrose, Teece) as we use, but using slightly different language.*

Conclusions: as the authors point out in page 9, “Despite cost reduction is the most frequent strategy of firms in economic crisis, especially in the short term; Some authors debate its effectiveness”; it would be interesting to explore whether firms that opt for unrelated diversification during burst had a higher payoff in terms for example of market value or other key performance indicators during booms. Provide additional information about the performances of these firms after a crisis, conditional on their diversification strategies, would strength the managerial and practical implications of the authors’ study, which are not included in the current version.

*These are interesting questions but are beyond the scope of this paper and would require a separate study.*
