# Supplementary material for: Related adjustment of firm production after demand shocks
Source: PLoS One. 2023 Jan 18;18(1):e0280461. doi: 10.1371/journal.pone.0280461 (PMC9847954; doi:10.1371/journal.pone.0280461)
Supplement: Attachment — Submitted filename: Response to the Reviewers Plos1_14122022.docx [file pone.0280461.s002.docx]

**Response to the Reviewers**

Dear Editor and Reviewers,

We appreciate the opportunity to revise and resubmit our paper “Related and unrelated firm diversification in crisis and in prosperity”. We are thankful for the comments of the reviewers that enabled us to develop our paper more solid theoretically and empirically. We are addressing each of the concerns and comments raised step by step in the followings.

Reviewer #1: The authors have properly addressed almost all issues raised in the previous referee reports. I would add just one concern and a suggestion.

My concern is that the authors should be more cautious in interpreting findings. They too often interpret them in terms of 'impact', and they explicitly state that they are sure to rule out endogeneity by just adding lagged values of regressors. I understand that this point was raised by the other referee. From an econometric perspective, lagged values in a panel data setting are just a naive solution to endogeneity, perhaps just a first step to address simultaneity (which is one possible story of endogeneity). Yet, the underlying economic relationships that the authors are testing are complex, and I would not rule out possible 'reverse causality' issues or a few omitted variable bias. The triad market structure/financial constraints/demand shocks has more than one possible direction of causality, as scholars of industrial organization or corporate finance could well explain. Eventually, my suggestion is to stay as much as possible with an interpretation in terms of correlations.

*Thank you. Accepting the reviewer's suggestion, we have restated our conclusions.*

Reviewer #2: I have read the paper and I am happy with some of the changes the authors have implemented. I feel that the paper is going towards a more convincing path. Nevertheless, I do not find all the concerns were addressed satisfactorily and a few minor issues have emerged in the new version. I have few additional comments and suggestions.

- Terminology: I appreciate that the authors have revised some terminology in the manuscript using demand shocks, however there is lack of consistency to this terminology along the full paper which requires a more in depth rephrase of the sentences and reconceptualization along the entire paper (in some passages there is still reference to differences between crisis and prosperous times, or to the use of different synonyms.) I would suggest the authors to define exactly what they mean with economic crisis and stick to their definition of crisis/demand shock along the entire paper (they are going in that direction but needs some more editing).

*Thank you. Reading the manuscript in this light, it became apparent that in the previous version the arguments about crises and demand shocks were mixed. So we cleared this up by concentrating on demand shocks, and mentioning the crises only as a specific case of negative shocks in the corresponding section of the literature (2.3.)*

- Type of burst: the type of burst or different types of crises/demand shocks raised in the previous round was not addressed satisfactory. I feel the authors have just added one sentences but the differences could be better described.

*In the previous round we were struggling finding proper solution. As we identify from the heterogeneity over thousands of products, they have turned out to be way too many to create meaningful visualizations, and picking random examples just felt arbitrary. Still, it is absolutely a valid claim to give the reader the feeling about the heterogeneity and the related economic trends here. Accordingly, we added the following paragraph.*

*“The investigated period covers interesting heterogeneity of demand shocks across product markets and firms. Trade liberalization opened the Hungarian industrial market in the 1990s and has concluded after the country joined the EU in 2004. This opening hit some of the traditional industries. For example, a significant decline was observed in the food industry due to the adverse effect of EU agricultural agreements, and in the and textile industry due to a reallocation mechanism that destroyed less productive firms (Békés-Halpern-Muraközy 2011, Harasztosi 2011) and to a significant increase of the minimal wage (Harasztosi and Lindner 2019). The period after EU accession is associated with increasing foreign direct investment, gross value added, and export orientation, which is interrupted in 2008-2010 by the global financial crisis that also hit industries to a different extent. After the financial crisis, a re-industrialization can be observed that is selective across industries. Transport equipment sector grew both in terms of employment and value-added, however, computers and electronics have decreased (Lengyel 2011, Lengyel et al 2017).”*

- Financial distress: I appreciated that the authors have added a variable that may capture the financial distress of the firms in their sample, and for consistency reasons I would like to see the full model in table 3. In the current version the authors argue that results are not altered therefore for consistency would be nice to report the full model.

*Thank you for the comment. We added that as Column 3 of Table 3.*

- Additional statistics: in the previous round I suggested to include some statistics about the firms in the sample. I appreciate the efforts of the authors in addressing this suggestion, however it is not fully clear to me whether the statistics come from Eurostat or from their sample. They argue that the PRODCOM database includes approximately 7000 firms but in the following paragraph of Section 3.1. they indicate that there were 550 firms operating in the observed period based on Eurostat. I would better clarify this point, where the statistics come from and so forth.

*Thank you. We reorganized the corresponding two paragraphs and added more details to provide the readers a clearer overview on the process and the sources of data.*

Moreover, although the authors do not have firm age, they could probably retrieve it from the date of incorporation, if available.

*Thank you. We finally obtained the date of incorporation from another data source. (Balance sheet data from the national tax authority) that we could merge to the balance sheets that we had from the statistical office, so we could include firm age to Table 1.*

- Theory: I also appreciate the efforts in adjusting the theory. However, I think that the introduction and theory part could be further strengthen by problematize more the topic of research. This is also linked to the fact that the results that firms opt for related products, especially those that are financially distressed, is not surprising per se. Therefore, I would suggest to address more in depth the theory and the empirics in order to create a sort of tension or to problematize better this interesting issue.

*Thank you. Now we have re-written the introduction to focus better on our contribution to the literature. We have also streamlined the theory, along your previous comment on the terminology, as we found that it was not only a terminology issue. We also added two papers, you recommended in the previous revisions (Aghion & Saint-Paul; Steenkapm & Fang) to improve the theory.*

I also noticed that in some of the theory, the authors make express reference to how the theory would adapt in case of small firms. Extending the discussion to how the mechanisms derived from the theory cited in the manuscript would adapt in the context of small firms make sense if the sample is mostly based on small. If this is the case, I would suggest to adapt Authors prediction based also on how the mechanisms and theory they describe would change, the authors partially do so for example at the end of Section 2.2.

*Thank you. In fact, this is true for our sample, the majority of the firms are small, falling into the 10-50 employee category. This is however just the consequence of the distribution of firms, which we did not want to restrict further, beyond that is applied in the PRODCOM generally.*

*In this version we added a sentence along the specificities of small firms to Section 3.3. too, in addition what was already present in section 2.2. However, we did not want to elaborate this line of argument much further, because that would eventually lead to developing specific hypotheses for small versus large firms, where we would not want to drive this paper for both theoretical and practical reasons. On the theoretical side we observe that those papers referring to small firms have only small firms in their samples, and do not make comparisons by large ones, therefore we cannot easily develop consistent hypotheses to differentiate them. On the practical side we think that the current empirical examination having two shock mechanisms, the interaction of financial exposure, and the two adjustment channels of dropping and adding products is rich enough, so it would have been too much if we added a further interaction of firm size.*

- Editing: I noticed that some of the new passages are a bit fragmented and there are some typos. Along this line, I noticed that in the abstract, intro and theory, the authors refer to the coherence measure using the term cohesiveness. As suggested in the first comment, also in this case, I would stick to the term coherence in order to make it consistent with the empirics. In table 3 I would clarify whether the variables are at t-1, I think so based on your equation N. 9, if so I would suggest adapt the table.

*Thank you, we reviewed the text, got rid of the cohesiveness term and edited the tables to be consistent with the equations.*
